# Lateral Medullary Syndrome Due to a Hypoplastic Vertebral Artery

**DOI:** 10.7759/cureus.30463

**Published:** 2022-10-19

**Authors:** Albert M Chung, Lisa Sovory

**Affiliations:** 1 Neuroradiology, California University of Science and Medicine, Colton, USA; 2 Neurology, California University of Science and Medicine, Colton, USA

**Keywords:** vertebrobasilar insufficiency, posterior circulation stroke, wallenberg syndrome, lateral medullary syndrome, vertebral artery hypoplasia

## Abstract

Vertebral artery hypoplasia is an uncommon congenital anatomical variation. Currently, no standard criteria exist for evaluating vertebral arteries as being hypoplastic based on vessel luminal diameter or volume flow. There is debate on the clinical significance of these variants and their relevance as an independent risk factor for posterior circulation strokes. We report a case of a 59-year-old male presenting with lateral medullary syndrome in the setting of a left hypoplastic vertebral artery with absence of atherosclerotic or thrombotic involvement.

## Introduction

Vertebral artery hypoplasia (VAH) is a congenital anatomical variation that has been frequently characterized as having a vertebral artery diameter <2.2 mm or vertebral artery volume flow <30-40 mL/min on duplex ultrasound; however, there is no clear consensus on these values [1]. Some authors purport that these anatomic variants are asymptomatic and do not predispose to vertebrobasilar insufficiency [2-4]. Conflicting studies have demonstrated large differences in the prevalence of VAH with one study reporting 11.6% in the general population versus 35.2% in ischemic stroke patients with a preference for the right vertebral artery [5]. Left-sided variations account for approximately 10% of patients with VAH and often result in its termination into the posterior inferior cerebellar artery.

Mechanisms that support the pathogenicity of VAH are controversial and include embolism, as is frequently the case for infarcts secondary to internal carotid artery plaque formation, and reduced vertebral artery flow volume [6-7]. Although it has been proposed that the dominant vertebral artery can provide appropriate compensation, Doppler ultrasound studies have demonstrated higher resistivity indices for the dominant vertebral artery due to increased collateral flow. This results in vascular compromise for both vertebral arteries and thus increases the risk of posterior circulation strokes.

Here, we report a case of a 59-year-old Hispanic male diagnosed with lateral medullary syndrome secondary to VAH with patent blood flow on computed tomography (CT) and without significant atherosclerotic or thrombotic events.

## Case presentation

A 59-year-old Hispanic male was admitted to a teaching hospital in San Bernadino County, California, on April 11, 2022, with a two-day history of dizziness, swaying towards his left side, left-sided facial droop, dysphagia, and dysarthria. His past medical history included a two-year-old ischemic stroke in the left parietal-temporal region, hypertension, and insulin-dependent diabetes mellitus on aspirin, simvastatin, lisinopril, metformin, glipizide, insulin lispro, and insulin glargine. Vitals recorded in the emergency department revealed a blood pressure of 142/87 mm Hg, a pulse of 72 beats per minute, a temperature of 37.1 °C, and a respiratory rate of 15 breaths per minute. The patient was admitted to the stroke unit with telemetry, and the neurology team was consulted in the morning. His neurological examination revealed bilateral nystagmus on horizontal conjugate gaze, vertigo, palatal weakness, dysarthria, dysphagia, left-sided ptosis, left-sided facial droop, left-sided dysmetria during finger-nose testing, and left-sided ataxia during heel-shin testing.

**Figure 1:**
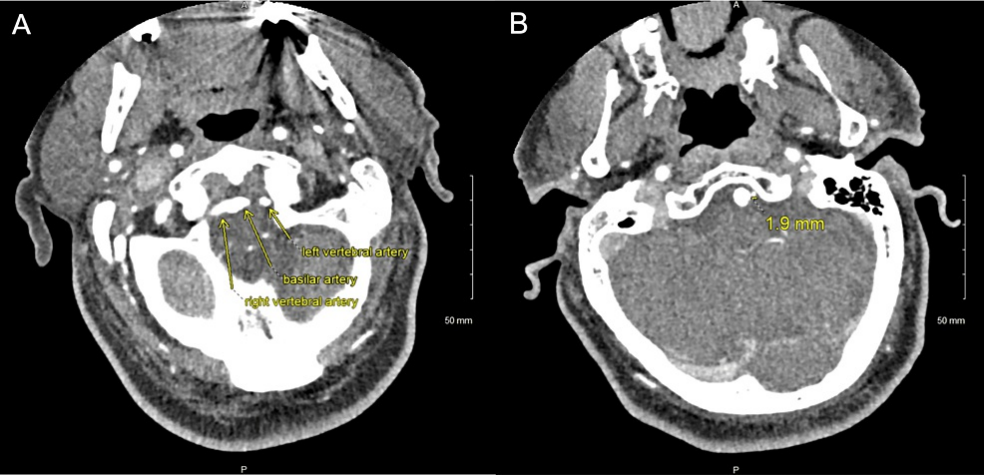
CT angiography of the head and neck

**Figure 2:**
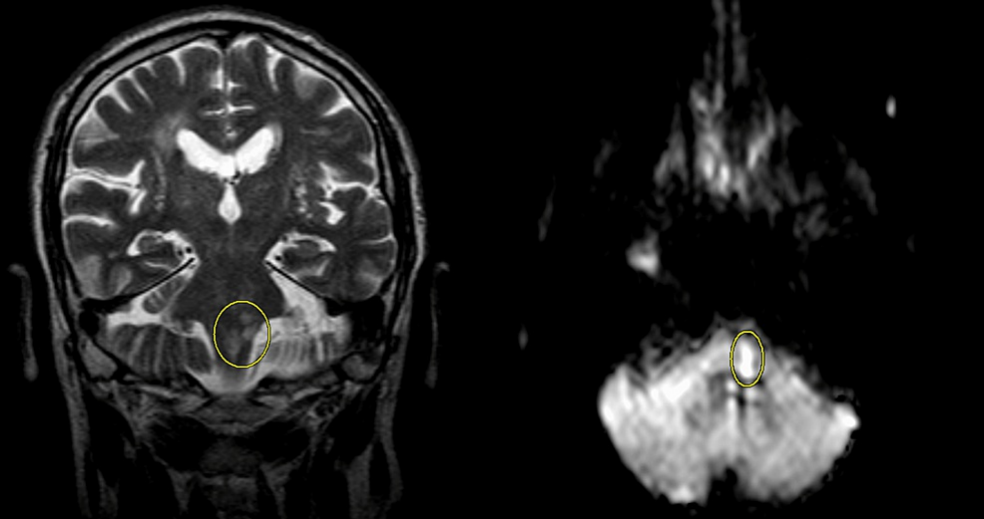
MRI of the brain without contrast showing restricted diffusion

CT angiography (CTA) of the head and neck with contrast was performed shortly after admission and revealed no hemodynamically significant stenosis, atherosclerotic plaques, or thrombus; however, the patient was noted to have a rare congenital anatomical variation of his left vertebral artery that did not converge with the right vertebral artery at the base of the basilar artery (Figure 1A). The diameter of the left vertebral artery was measured as 1.9 mm (Figure 1B). Normal flow voids were noted in the major cerebral arteries and veins. Brain MRI without contrast was performed on the same day and showed restricted diffusion with T2 hyperintensity in his left brainstem at the pontomedullary junction with extension into the medulla (Figure 2). Overall, the clinical picture and MRI findings were consistent for lateral medullary syndrome due to an acute ischemic infarct in a region of the brainstem supplied by the left vertebral artery. Based on clinical acumen and preserved flow voids with the absence of a thrombus/embolus on imaging, the left vertebral artery was determined to be sufficiently patent and unlikely to cause vertebrobasilar insufficiency. Further work-up was initiated to rule out other causes of the acute ischemic stroke.

Given the presence of a stroke, dual antiplatelet therapy was started with aspirin 325 mg and clopidogrel 75 mg daily for a duration of three months. Permissive hypertension was allowed for 24 hours before the initiation of normotensive blood pressure control. Tight glycemic control via insulin sliding scale was initiated given that the patient’s hemoglobin A1c (HbA1c) was 8.5%. The fasting lipid panel revealed a mildly decreased high-density lipoprotein (HDL) level. EKG was unremarkable for atrial fibrillation. On day 2, the patient underwent a transthoracic echo with a bubble study that was unremarkable for a thrombus or structural defects that may transmit a paradoxical embolus to the cerebrovascular system. On day 3, a fluoroscopic esophageal barium swallow with video and speech was performed and was negative for any structural causes of dysphagia and dysarthria. Throughout the duration of his in-patient admission, his symptoms did not improve or resolve.

## Discussion

Due to the high prevalence of VAH in the general population, the clinical significance of VAH may be often overlooked as a significant risk factor for vertebrobasilar insufficiency. However, patients with hypoplastic vertebral arteries have a higher probability of developing strokes, in particular the posterior circulation compared to the anterior circulation [8].

Our patient presented with a rare anatomical variation of his vertebral artery that was hypoplastic on his left vessel and did not contribute to the basilar artery. Given the results of our additional work-up, it is highly unlikely that the acute ischemic stroke was secondary to an atheromatous embolus or clot but rather due to the compromised vertebrobasilar blood flow. Our clinical observations and MRI images consistent with an acute ischemic stroke in the left brainstem conflict with volume flow cutoffs for VAH [9]. Although not widely considered standard of care, performing color Doppler sonography could be a consideration in patients with brainstem strokes as studies have shown measurements of the peak systolic velocity ratio to be the most accurate predictor of stenosis in vertebral arteries instead of vessel diameter [10-11].

Some authors believe that the pathologic relationship between VAH and lateral medullary infarcts may be due to the curvature of the basilar artery. Hong et al. demonstrated that the diameter of the vertebral artery is the only independent predictor for moderate-to-severe angulations of the basilar artery after adjusting for demographics, radiological findings, and hemodynamic variables [12]. They proposed that smaller vertebral artery diameters result in basilar artery dolichoectasia secondary to asymmetric wall tension. This results in morphological and structural adaptation due to weakening of the tunica intima. This causes the basilar artery to lose its original conformation and elongate to withstand the higher wall pressure. Overall, the pathologic mechanism of VAH may arise from increased thrombogenicity at the site of basilar artery curvature.

## Conclusions

Hypoplastic vertebral arteries may predispose one to posterior circulation strokes due to the small vessel diameter, reduced arterial blood flow, or, indirectly, by increasing the curvature of the basilar artery. We suggest that further research should be conducted to study the risk-benefit ratio of screening for VAH on duplex ultrasound, particularly in patients with risk factors for vascular insufficiency.
